# Environmental Lead Exposure, Catalase Gene, and Markers of Antioxidant and Oxidative Stress Relation to Hypertension: An Analysis Based on the EGAT Study

**DOI:** 10.1155/2015/856319

**Published:** 2015-02-22

**Authors:** Jintana Sirivarasai, Sukhumpun Kaojarern, Suwannee Chanprasertyothin, Pachara Panpunuan, Krittaya Petchpoung, Aninthita Tatsaneeyapant, Krongtong Yoovathaworn, Thunyachai Sura, Sming Kaojarern, Piyamit Sritara

**Affiliations:** ^1^Graduate Program in Nutrition, Faculty of Medicine Ramathibodi Hospital, Mahidol University, Bangkok 10400, Thailand; ^2^Cardiovascular and Metabolic Center, Faculty of Medicine Ramathibodi Hospital, Mahidol University, Bangkok 10400, Thailand; ^3^Office of Research Academic and Innovation, Faculty of Medicine Ramathibodi Hospital, Mahidol University, Bangkok 10400, Thailand; ^4^Department of Medicine, Faculty of Medicine Ramathibodi Hospital, Mahidol University, Bangkok 10400, Thailand; ^5^Research and Development Institute, Kasetsart University, Bangkok 10900, Thailand; ^6^Health Office, Electricity Generating Authority of Thailand, Nonthaburi 11130, Thailand; ^7^Department of Pharmacology, Faculty of Science, Mahidol University, Bangkok 10400, Thailand; ^8^Occupational and Environmental Toxicology Center, Faculty of Medicine Ramathibodi Hospital, Mahidol University, Bangkok 10400, Thailand

## Abstract

Lead has been linked to the development of hypertension via oxidative stress. Catalase plays an important role in the disposal of hydrogen peroxide in erythrocyte and its activity was determined by CAT gene. The aims of this study were to investigate (1) the association between blood levels of antioxidant markers such as catalase, superoxide dismutase, glutathione, glutathione peroxidase, oxidative stress-marker (malondialdehyde), and blood lead level and (2) the influence of genetic polymorphism of CAT gene (rs769217) on change in blood pressure in general population of EGAT study project. This is a cross-sectional study of 332 normotensive, 432 prehypertensive, and 222 hypertensive male subjects. Hypertensive subjects had significantly higher blood lead level (5.28 *μ*g/dL) compared to normotensive (4.41 *μ*g/dL) and prehypertensive (4.55 *μ*g/dL) subjects (*P* < 0.05). These significant findings are also found in MDA levels. Moreover, individuals with TT genotype in hypertensive group had significantly higher blood lead and MDA levels (6.06 *μ*g/dL and 9.67 *μ*mol/L) than those with CC genotype (5.32 *μ*g/dL and 8.31 *μ*mol/L, *P* < 0.05). Our findings suggested that decreased blood catalase activity in this polymorphism together with low level lead exposure induced lipid peroxidation may be responsible for hypertension.

## 1. Introduction

General population may be exposed to lead through various sources such as dietary contamination (via food chain and lead releasing from food containers or ceramic glaze), public water supplies contamination, herbal remedies, and manufacturing byproducts such as E-waste recycling, manufacture of batteries, sheet lead, solder, brass, and bronze plumbing, radiation shields, circuit boards, and military equipment [1]. Lead exposure occurs mainly through the respiratory and gastrointestinal tracts. Approximately 30–40 percent of inhaled lead is absorbed into the bloodstream. Gastrointestinal absorption varies depending on nutritional status (i.e., iron or calcium deficiency) and age. Once absorbed, 99 percent of circulating lead is bound to erythrocytes for approximately 30–35 days (estimating about 1% absorbed lead is found in plasma and serum) and is dispersed into the soft tissues, including renal cortex, liver, lung, brain, teeth, and bones [1].

Since bone accounts for more than 94% of the adult body burden of lead, bone lead level by K-X-ray fluorescence represents lead content in the cortex of tibia and the patella trabecular [2]. This measurement is an indicator of cumulative lead exposure and is particularly relevant to the elderly in whom elevated bone lead concentrations may represent chronic toxicity [3]. Measuring blood lead is the most commonly accepted and verifiable biomarker for lead exposure. This assessment, by industrial hygienist, was used both in current and past environmental lead exposures to quantify the intensity of the exposure [4]. In the blood stream, lead circulating is mobile whereas lead in bone is stored. Mobile lead exerts adverse effects on human body. Under conditions of more or less constant and prolonged exposure, an individual's blood lead level reflects the quantity of biological active forms of lead in their body [5]. A large number of reports revealed positive correlations between blood lead and detrimental effects on the central nervous, hematopoietic, renal, immune, and cardiovascular systems [6].

Hypertension is a multifactorial condition associated with both environmental and genetic factors. For environmental risk factors include dietary, lifestyle, obesity, and some toxicants, lead is one of the candidate metals which can be linked to the development of hypertension [7, 8]. Numerous human and animal studies found a causal relationship between low-level lead exposure and hypertension. Some evidences indicated that oxidative stress played a significant role in the etiology of lead-induced hypertension [8]. Oxidative stress is described as a physiological stage in which antioxidant defense is inadequate to detoxify the reactive oxygen species (ROS). This oxidative process results in the damaging of essential biomolecules such as protein, lipid, and DNA. Overproduction of ROS is demonstrated in lead-induced oxidative stress. Previous experimental studies revealed that lead could promote ROS production in kidney and cardiovascular tissues [9, 10]. In addition, lead influenced cell membrane alterations, such as lipid component, membrane integrity, permeability, and function, finally leading to lipid peroxidation [11, 12].

The most common group of indices used to assess oxidative stress is that of peroxidation products of lipids, usually polyunsaturated fatty acids, which are susceptible to attack by free radicals. All these products of degradation and decomposition are used in assessing oxidative stress, including hydroperoxides, F2-isoprostanes, and malondialdehyde (MDA) [13]. MDA is the principal and most studied product of polyunsaturated fatty acid peroxidation. This aldehyde is a highly toxic molecule and should be considered as more than a marker of lipid peroxidation [14]. Derivatization of MDA with thiobarbituric acid (TBA), as MDA-TBA adduct, is a wildly used method to monitor the level of lipid peroxidation in biological sample. The HPLC with fluorescence detection significantly improved the specificity and overcame overestimation of the MDA-TBA adduct, as indicated by much more homogenous results obtained in various publications [15]. By measurement of F2-isoprostanes, TBA-MDA adduct, or lipid hydroperoxides, there were some reports that showed correlations between TBA-MDA adduct and F2-isoprostanes or lipid hydroperoxides [12].

Another mechanism of lead-induced oxidative stress is the effect on antioxidant defense systems of cells. Lead exhibits a high affinity for sulfhydryl (SH) groups and can interfere with antioxidant activities by inhibiting functional SH groups in several enzymes such as superoxide dismutase (SOD), catalase (CAT), glutathione peroxidase (GPx), glucose-6-phosphate dehydrogenase (G6PD), and ALAD [16]. A large number of researches were conducted to further understand the imbalance between antioxidant and oxidant stages with risks of chronic diseases, especially in the field of genetic variations of antioxidant enzymes [17–19]. Catalase is a well-known antioxidant enzyme that plays a role in the conversion of H_2_O_2_ to H_2_O and O_2_ [20]. The CAT gene is located in chromosome 11p13 and consists of 13 exons. The C111T polymorphism of this gene, in exon 9 (rs769217), is responsible for alteration in its activity [21]. To our knowledge, data related to the influence of CAT C111T polymorphism on antioxidant system/oxidative stress and hypertension in environmental lead exposure are very limited. Moreover, this is the first study in Thai population that emphasizes on biomarkers of lead exposure and of susceptibility with CAT gene. The aims of the present study were to investigate (1) the association between blood levels of antioxidant markers such as CAT, SOD, GPx, glutathione (GSH), MDA, and blood lead level and (2) the possible influence of CAT polymorphism on change in blood pressure among general population.

## 2. Materials and Methods

### 2.1. Study Population

The Electric Generating Authority of Thailand (EGAT) study was the first cohort study of chronic disease in Thailand, originally designed in 1985 (known as EGAT 1), and mainly covered multidisciplinary researches related to cardiovascular disease (CVD) risks such as nutrition and toxicology. The 986 male subjects were participants in the third survey of EGAT 2 in 2009 (the first survey started in 1998 and the second survey in 2003). This study was approved by the Committee on Human Rights Related to Researches Involving Human Subjects, Faculty of Medicine Ramathibodi Hospital, Mahidol University, Thailand. All participants completed a self-administered questionnaire and underwent a physical examination and performed laboratory analysis, including tests for diabetes and liver and kidney diseases [22]. Toxicological profile of heavy metals and genetic analysis were determined. Ten milliliters of blood was collected by venipuncture into EDTA and heparinized tubes from each subject and immediately centrifuged at 2000 g. Buffy coat, erythrocytes, and plasma were separated and stored at −20°C until genotyping analysis and biochemical measurements were performed.

According to the Joint National Committee 7 (JNC 7), hypertension was defined as systolic blood pressure (SBP) ≥140 mmHg or diastolic blood pressure (DBP) ≥90 mmHg, prehypertension was defined as SBP 120–139 mmHg or DBP 80–89 mmHg, and normal blood pressure was defined as SBP <120 mmHg and DBP < 80 mmHg [23]. Based on this criterion, participants were classified into 3 groups: 332 normotensive, 432 prehypertensive, and 222 hypertensive subjects.

### 2.2. Determination of Blood Lead

Blood lead concentration was measured by graphite furnace atomic absorption spectrometry (GFAAS) with Zeeman background correction. The analytical procedure was based on the method described by Subramanian and Meranger [24]. The measurement was calculated as micrograms per deciliter (*μ*g/dL) and expressed by means of total blood lead. The intra-assay coefficients of variation (CV) ranged from 2.8 to 5.9% and interassay CV ranged from 3.2 to 6.4%.

### 2.3. Determination of Glutathione by the DTNB Method

Whole blood (0.1 mL) was added to distilled water (1.9 mL) together with 3 mL of precipitating solution (1.67 g glacial metaphosphoric acid, 0.2 g disodium ethylenediaminetetraacetic acid, EDTA, and 30 g sodium chloride). Then, the filtrate (0.5 mL) was added to 0.3 M phosphate buffer, pH 6.4 (2 mL). Finally, 1 mM DTNB (0.25 mL) was added, mixed well, and the absorbance was read at 412 nm within 4 min [25]. The intra-assay and interassay CV were 4.3% and 6.0%, respectively.

### 2.4. Determination of Catalase Activity

Catalase activity was measured by the decrease in absorbance at 240 nm due to H_2_O_2_ consumption, according to Aebi method [26]. Hemolysate (0.1 mL) was added to cuvette containing 1.9 mL of 50 mM phosphate buffer (pH 7.0). Enzymatic reaction was started by the addition of 1.0 mL of freshly prepared 30 mM H_2_O_2_. The rate of decomposition of H_2_O_2_ was measured by spectrophotometer from changes in absorbance at 240 nm. Activity of catalase was expressed as U/gHb. The intra-assay and interassay CV were 3.2% and 4.7%, respectively.

### 2.5. Determination of SOD Activity

Superoxide dismutase (SOD) activity was measured by the method of Winterbourn et al. [27]. This method used nitroblue tetrazolium (NBT) as indicator and riboflavin as superoxide-generating system. Result was expressed as unit of SOD per gram of Hb. One unit is defined as the amount of enzyme causing half the maximum inhibition of NBT reduction. The intra-assay and interassay CV were 3.6% and 5.5%, respectively.

### 2.6. Determination of GPx Activity

GPx activity was assayed by method of Beutler [28], using t-butyl hydroperoxide (t-BuOOH) and glutathione (GSH) as substrates, and this was followed by measuring the oxidation of NADPH at 340 nm at 37°C with a spectrophotometer in the presence of glutathione reductase. The reaction mixture consisted of 0.5 mM EDTA, 0.1 M Tris-HCl (pH 8.0), 2.0 mM NADPH, 2 mM GSH, 1 U glutathione reductase, and hemolysate in a total volume of 0.99 mL. The enzymatic reaction was initiated by the addition of 10 *μ*L t-BuOOH. One unit of GPx activity is defined as the amount of enzyme that oxidizes 1 *μ*mol NADPH/min. GPx activity was expressed as U/gHb. The intra-assay and interassay CV were 4.6% and 6.8%, respectively.

### 2.7. Determination of MDA

MDA was determined using an HPLC method with fluorescence detector, as described by Khoschsorur et al. [29]. Detection limit was 0.25 *μ*mol/L and this method exhibited a linear response of MDA in a range of concentration from 1.50 to 15.0 *μ*mol/L and calibration curve presented high correlation coefficient (*r* ^2^ > 0.90, *P* = 0.001; *n* = 10). The intra-assay and interassay CV were 3.9% and 4.7%, respectively.

### 2.8. Genotyping Assay

The genomic DNA was extracted from lymphocytes by a modified salting-out procedure [30] and frozen at −20°C until analysis. The genetic polymorphism of* CAT (rs769217) *was performed by real-time polymerase chain reaction (real-time PCR) according to the method of TaqMan SNP Genotyping Assays on an ABI 7500 instrument (Applied Biosystems, Foster City, CA, USA), in 96-well format. The TaqMan Assay included the forward target-specific polymerase chain reaction (PCR) primer, the reverse primer, and the TaqMan MGB probes labeled with 2 special dyes: FAM and VIC. The concentrations of probes were 0.04 *μ*M. Amplification of 20 ng of DNA was performed during 40 cycles in a reaction volume of 10 *μ*L. TaqMan Universal PCR Master Mix was used for analysis. Thermocycling conditions were 95°C for 15 seconds, followed by 60°C for 1 minute. Information of specific probe and primers is available on the National Cancer Institute's SNP500 database web page at http://snp500cancer.nci.nih.gov/ [31]. Quality control procedure included repeat genotyping of at least 10% of DNA samples. On every single 96-well microtiter plate, we include negative control in the form of water. The percentage of successful rate for 95 cases and negative control was 100 and this assay was good allelic discrimination among individuals. For overall SNP genotyping assay, the reported error rate for 983 cases was 0.33% which involved DNA extracts of poor quantity and quality.

### 2.9. Statistical Analysis

Statistical analyses were carried out using the SPSS 16.0 for window software (SPSS, Inc., Chicago, IL). Most of the study parameters were presented as mean ± SE. Because of skewed distribution, lead level was transformed to normal distribution and expressed as geometric mean ± SE. The comparisons between variables were examined by Student's *t*-test and analysis of variance (ANOVA). Genotype distribution was analyzed with *χ* ^2^. Pearson's correlation was performed to determine the strength of the association between blood lead and other significantly correlated parameters. A *P* value of 0.05 was used as the criterion for statistical significance.

## 3. Results

The clinical characteristics and biochemical profile in normotensive, prehypertensive, and hypertensive groups are presented in Table 1. Mean age for subjects with hypertension was comparable to those with prehypertension and normotension. More than 50% of the three groups were in the range of 40–55 years. The control group (23.58 kg/m^2^) has significantly lower BMI than prehypertensive (25.09 kg/m^2^) and hypertensive subjects (26.26 kg/m^2^, *P* < 0.05). SBP and DBP in hypertensive group (144.9 and 93.7 mmHg) also showed statistically higher than those in the prehypertensive (126.1 and 80.94 mmHg) and control groups (110.2 and 70.3 mmHg, *P* < 0.05). No significant differences were found with respect to distributions of alcohol consumption and smoking status among three groups. Furthermore, subjects with prehypertension and hypertension had statistically higher blood levels of triglyceride, fasting glucose, creatinine, and uric acid than the normotensive subjects.

**Figure 1:**
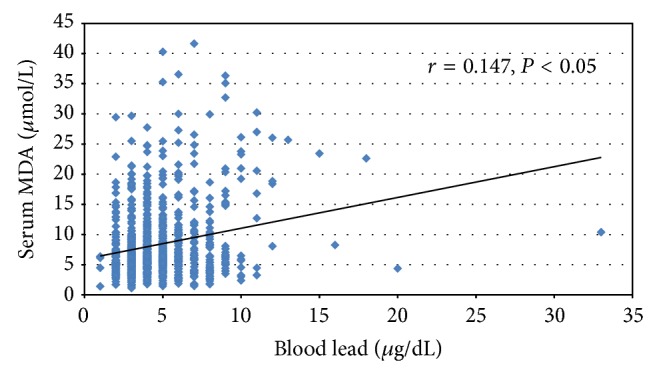
Association between blood lead and serum MDA levels in the study population.

**Figure 2:**
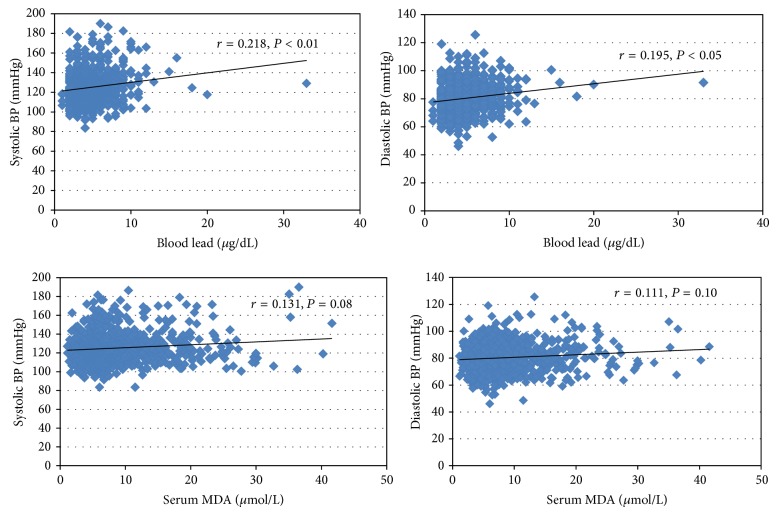
Association between blood lead, serum MDA, and SBP and DBP in the study population.

As shown in Table 2, there was a significant increase in blood lead and MDA levels among the three groups, but statistical changes in antioxidant parameters were not found. Hypertensive subjects had significantly higher blood lead level (5.28 *μ*g/dL) compared to normotensive (4.41 *μ*g/dL) and prehypertensive (4.55 *μ*g/dL) subjects (*P* < 0.05). In addition, blood MDA level in hypertensive group (9.64 *μ*mol/L) was statistically higher than those in prehypertensive (8.02 *μ*mol/L) and normotensive groups (8.23 *μ*mol/L, *P* < 0.01). There were no significant differences in the means of CAT, SOD, GPx, and GSH among the three groups. Our results illustrated that SBP (*r* = 0.218, *P* < 0.01), DBP (*r* = 0.195, *P* < 0.05), and MDA concentrations (*r* = 0.147, *P* < 0.01) were positively correlated with blood lead level (Figure 1). There was no significant association between blood MDA and SBP or DBP (*r* = 0.131 and *P* = 0.08 and *r* = 0.111 and *P* = 0.10, resp.) (Figure 2).

**Figure 3:**
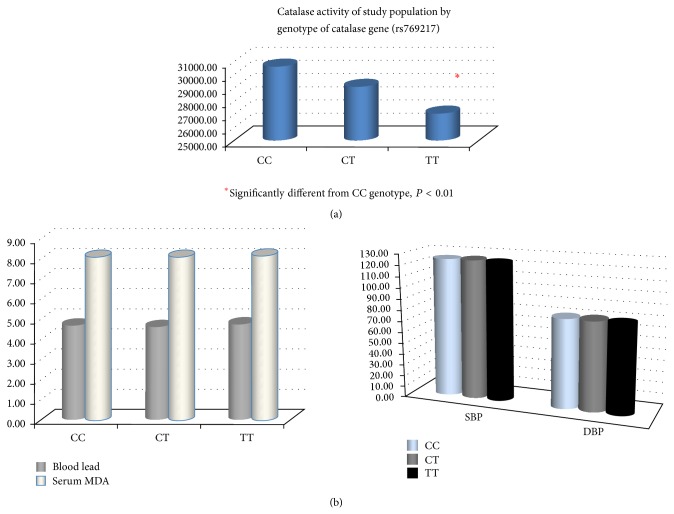
Genetic polymorphism of CAT gene (rs769217) and its activity (upper) and the comparison of blood lead, MDA, and BP of all study populations classified by genotype (lower).

Genotype frequencies of CAT C111T polymorphism in this study population were 32.0% for CC, 46.2% for CT, and 21.7% for TT. The genotype distribution among controls was according to the Hardy-Weinberg equilibrium (*P* = 0.28). Catalase activity was significantly lower in individuals with TT genotype (27044 U/gHb) compared to those with CT genotype (29103 U/gHb) or CC genotype (30625 U/gHb) (*P* < 0.01). However, the means of blood lead, MDA, SBP, and DBP of individuals with CC, CT, or TT genotypes did not reveal differences (Figure 3).

Table 3 illustrates the effect of CAT genotypes on biochemical profile, blood lead, and antioxidant and oxidative stress determinants in the three groups classified by CAT genotypes. All groups with CC, CT, and TT genotypes had no significant (*P* > 0.05) differences for lipid profiles and other biochemical parameters (FBS, Cr, and UA). Further analysis in the same fashion with oxidative stress and antioxidant defense found that there was significant change of catalase activity with higher activity in wild-type allele (32341 U/gHb) and lower activity in those with mutant allele (27423 U/gHb, *P* < 0.05) in controls. Similar (*P* < 0.05) blood catalase activities were found for CC, CT, and TT genotypes in prehypertensive and hypertensive subjects. In contrast, SOD activity in the hypertensive group showed different trend (2055 U/gHb for CC versus 2616 U/gHb for TT, *P* < 0.05). The CAT C111T significantly modified the effect of lead on MDA, as seen only in hypertensive group, in which individuals with TT genotype had significantly higher blood lead and MDA levels (6.06 *μ*g/dL and 9.67 *μ*mol/L) than those with CC genotype (5.32 *μ*g/dL and 8.31 *μ*mol/L, *P* < 0.05).

## 4. Discussion 

Exposure to low level of lead in environmental manner has been reported to cause dysfunction in many target organs. The overt toxicity of this metal may result from its potentially induced oxidative stress as seen by an increased prevalence of chronic kidney disease, cardiovascular disease, peripheral arterial disease, diabetes, hyperuricemia, or hypertension [7–9]. The positive correlation between blood lead level and SBP or DBP has been well documented. These associations have been reported in both occupational workers and population with low exposure to environmental lead. In this study, hypertensive group has higher blood lead level (5.28 *μ*g/dL) than controls (4.41 *μ*g/dL) (Table 2). In addition, the positive correlations between blood lead level, SBP, and DBP are shown in Figure 2. Skoczynska [32] suggested various cardiovascular mechanisms of the hypertensive effect by low doses of lead, including changes in metabolisms of catecholamines, increased activity of the central adrenergic system, decreased activity of ATP-ase, changes in transmembranal transport of ions, inhibition of Na+ secretion and increase in blood volume, increased plasma rennin activity, and enhanced free radicals generation. Moreover, our previous study revealed the relationship between the effect of lead exposure on inflammatory marker (high-sensitivity C-reactive protein, hs-CRP) and the adverse change in SBP [33].

Lipid peroxidation is a well-established mechanism of cellular injury and is used as an indicator of oxidative stress in cells and tissues. In addition, measurement of MDA is widely used as an indicator of lipid peroxidation. The findings of the present study also supported the evidence of lead-induced ROS generation which represented oxidative stress (Table 2 and Figure 1). There was an increase in the MDA levels in the hypertensive group (9.46 *μ*mol/L) in comparison to those in the control group (8.23 *μ*mol/L) (Table 2). In addition, MDA level showed a significant increase with blood lead level (Figure 2). However, there were no significant differences in GPx activity and GSH level between controls and prehypertensive or hypertensive group. Catalase and SOD activities showed significant changes in hypertensive subjects. The results indicated adaptive mechanism in case oxidative stress occurred in cell. Impairing these enzyme activities may lead to the excess condition of free radicals which can escape antioxidant defense system, contribute to alteration of nitric oxide bioavailability, and affect structure/function of endothelium, resulting in high blood pressure [34].

Catalase has a predominant role in the disposal of hydrogen peroxide in human erythrocytes. Deficiency of erythrocyte catalase causes increased hydrogen peroxide concentration. A few polymorphisms have been revealed for the catalase-encoding gene. Genetic variations of this gene can be responsible for the change of its activity and expression. The analyzed polymorphism in this population is the C111T polymorphism in exon 9 (rs769217). Yung et al. [20] revealed the difference in catalase activity between wild-type and mutant alleles. Allele frequencies for the C and T reported in our study (55% and 45%, resp.) were similar to those reported previously in Japanese (C allele: 48%; T allele: 52%) [35] and Korean (C allele: 60%; T allele: 40%) [36] studies. In contrast to some countries, the T alleles were less frequent in Hungry (28%) [37], USA (12%) [38], and UK (17%) [20]. Based on genetic determinant of enzyme activity, this study revealed that individuals with TT genotype of C111T polymorphism associated with low catalase activity compared to those with wild-type allele (Figure 3). These findings can be explained by the data in which this nucleotide change may cause slower transcription from the mutant allele than from the wild-type allele [37].

To our knowledge, the present study is the first research that investigates the effect of CAT polymorphism on blood lead and blood pressure among nonoccupational population. Statistical differences in mean enzyme activities by genotypes were detected. The C111T polymorphism has an effect on blood catalase activity in all study groups (Table 3) but, for the hypertensive group, SOD activity in mutant allele (2616 U/gHb) was significantly higher than the wild-type allele (2055 U/gHb, *P* < 0.05). As mentioned above, these enzyme activities were partially determined by genetic factors and further modified by other various factors, such as exercise, stress condition, dietary intake of antioxidant, and coexposures to some toxicants. Moreover, individuals with TT genotype in hypertensive group showed significantly higher blood lead and MDA level than those with CC genotype. Decreased blood catalase activity in this polymorphism together with low-level lead exposure induced lipid peroxidation may be responsible for hypertension.

This study has an important strength, because EGAT study is the first cohort study in Thailand (EGAT 2 started in 1998), focusing on chronic disease with a five-year time series of survey. Thus, it also provides the opportunity to reevaluate the relationship between lead exposure and blood pressure with the large sample size. A limitation of our study is that blood lead concentration, as a biomarker of exposure, is not an appropriate marker for cumulative exposure when compared to bone lead. However, in vivo K-X-ray fluorescence for bone lead measurement is expensive and impractical for large-scale population studies. In addition, previous studies have shown strong association between blood lead and bone lead [39, 40] and most epidemiological studies to date wildly used blood lead level for biomonitoring of environmental lead exposure.

In conclusion, the evidence in this study was sufficient to infer a causal association between lead exposure and elevated blood pressure. Our finding suggested that exposure to low lead level associated with oxidative stress and deficiency of the enzyme catalase may contribute to hypertension. Further studies related to other genes involving antioxidant enzymes or elements will elucidate the relationship between environmental lead exposure, oxidative stress, and adverse health outcomes. For public health implication, regulatory plan and health intervention program should be developed and implemented for reduction of lead exposure and health risks such as cardiovascular, kidney, and other diseases.

## Figures and Tables

**Table 1:** General characteristics and biochemical parameters of study population according to hypertension.

Variables	Normotension (*N* = 332)	Prehypertension (*N* = 432)	Hypertension (*N* = 222)
Age, yrs (AM ± SE)	51.58 ± 0.23	52.01 ± 0.20	52.96 ± 0.33
Age group, *N* (%)			
40–55 yrs	267 (80.4)	328 (75.9)	152 (68.5)
>55 yrs	65 (19.6)	104 (24.1)	70 (31.5)
BMI, kg/m^2^ (AM ± SE)	23.58 ± 0.16	25.09 ± 0.15^a^	26.26 ± 0.25^a^
Drinking status, *N* (%)			
Nondrinkers	125 (37.7)	165 (38.2)	67 (30.2)
Drinkers	207 (62.3)	267 (61.8)	155 (69.8)
Smoking status, *N* (%)			
Nonsmokers	247 (74.4)	342 (79.2)	156 (70.3)
Smokers	85 (25.6)	90 (20.8)	66 (20.8)
Biochemical tests (AM ± SE)			
Triglyceride, mg/dL	132.81 ± 4.04	165.78 ± 5.35^a^	191.47 ± 8.81^a,b^
HDL cholesterol, mg/dL	49.57 ± 0.57	48.74 ± 0.49	49.32 ± 0.071
LDL cholesterol, mg/dL	152.31 ± 2.14	148.49 ± 1.91	146.61 ± 2.72
Total cholesterol, mg/dL	228.55 ± 2.31	230.33 ± 2.09	233.02 ± 2.79
Fasting glucose, mg/dL	100.58 ± 1.35	105.91 ± 1.24^a^	112.65 ± 3.09^a,b^
Creatinine, mg/dL	1.10 ± 0.07	1.14 ± 0.01^a^	1.14 ± 0.01^a^
Uric acid, mg/dL	5.97 ± 0.06	6.40 ± 0.06^a^	6.61 ± 0.08^a,b^

^a,b^Significantly different from normotension and prehypertension, respectively, *P* < 0.05.

**Table 2:** Blood lead level, antioxidants, and oxidative stress determinants of study population, according to hypertension.

Variables	Normotension (*N* = 332)	Prehypertension (*N* = 432)	Hypertension (*N* = 222)
Blood lead, *μ*g/dL	4.41 ± 0.10	4.55 ± 0.09	5.28 ± 0.21^a,b^
Antioxidant biomarkers			
CAT, U/gHb	29966 ± 549	28543 ± 422	28084 ± 600
SOD, U/gHb	2417 ± 80	2365 ± 71	2363 ± 96
GPx, U/gHb	34.56 ± 0.85	34.92 ± 0.78	36.11 ± 1.14
GSH, mg/dL	31.42 ± 0.34	31.41 ± 0.32	31.34 ± 0.46
Oxidative stress biomarker			
MDA, *μ*mol/L	8.23 ± 0.34	8.02 ± 0.26	9.64 ± 0.45^a,b^

^a,b^Significantly different from normotension and prehypertension, respectively, *P* < 0.05.

**Table 3:** Biochemical tests, blood lead, and determinants of antioxidant and oxidative stress, classified by CAT genotypes among three groups.

		Normotension		Prehypertension				Hypertension	
	CC (*N* = 104)	CT (*N* = 145)	TT (*N* = 83)	CC (*N* = 139)	CT (*N* = 207)	TT (*N* = 86)	CC (*N* = 73)	CT (*N* = 104)	TT (*N* = 45)
Biochemical tests^*^									
Triglyceride, mg/dL	135.0 ± 7.7	129.6 ± 6.2	135.5 ± 7.1	161.0 ± 8.9	166.7 ± 8.1	171.0 ± 11.7	166.8 ± 9.8	206.9 ± 14.9	195.9 ± 20.7
HDL cholesterol, mg/dL	49.36 ± 1.04	49.91 ± 0.87	49.25 ± 1.14	50.37 ± 0.93	49.56 ± 0.66	48.91 ± 1.16	50.83 ± 1.32	48.70 ± 1.01	48.32 ± 1.48
LDL cholesterol, mg/dL	149.4 ± 4.2	156.1 ± 3.2	149.21 ± 3.6	149.3 ± 2.9	148.1 ± 2.9	147.1 ± 4.4	151.1 ± 4.9	145.5 ± 4.1	141.9 ± 5.3
Total cholesterol, mg/dL	225.8 ± 4.6	232.0 ± 3.4	225.7 ± 4.0	232.4 ± 3.3	229.2 ± 3.2	229.8 ± 4.7	235.1 ± 5.1	234.2 ± 4.3	226.9 ± 4.6
Fasting glucose, mg/dL	102.2 ± 2.9	99.94 ± 2.06	99.91 ± 1.76	105.4 ± 2.1	106.1 ± 1.9	106.4 ± 2.6	104.6 ± 2.1	119. ± 5.9	109.1 ± 5.5
Creatinine, mg/dL	1.10 ± 0.01	1.11 ± 0.01	1.08 ± 0.01	1.11 ± 0.01	1.15 ± 0.01	1.15 ± 0.02	1.15 ± 0.02	1.13 ± 0.02	1.14 ± 0.02
Uric acid, mg/dL	5.95 ± 0.12	5.94 ± 0.09	6.03 ± 0.13	6.51 ± 0.09	6.31 ± 0.09	6.46 ± 0.14	6.57 ± 0.13	6.60 ± 0.13	6.72 ± 0.19
Blood lead, *μ*g/dL^**^	4.32± 0.17	4.51 ± 0.16	4.36 ± 0.21	4.71 ± 0.16	4.42 ± 0.14	4.59 ± 0.21	5.32 ± 0.31	5.38 ± 0.22	6.06 ± 0.41^a^
Antioxidant determinants^**^									
CAT, U/gHb	32341 ± 1017	29717 ± 766^a^	27423 ± 1129^a,b^	29621 ± 563	28014 ± 425	26741 ± 498^a^	28745 ± 765	28012 ± 568	26104 ± 925^a^
SOD, U/gHb	2301 ± 133	2571 ± 136	2295 ± 135	2286 ± 114	2436 ± 107	2321 ± 161	2055 ± 151	2469 ± 131	2616 ± 265^a^
GPx, U/gHb	34.31 ± 1.63	36.19 ± 1.33	32.03 ± 1.42	36.05 ± 1.41	34.58 ± 1.14	33.91 ± 1.74	37.65 ± 1.87	34.51 ± 1.59	37.32 ± 2.97
GSH, mg/dL	31.16 ± 0.62	32.05 ± 0.49	30.64 ± 0.73	31.46 ± 0.55	31.76 ± 0.48	30.50 ± 0.64	30.75 ± 0.69	30.97 ± 0.70	33.17 ± 1.12
Oxidative stress determinant^**^									
MDA, *μ*mol/L	7.95 ± 0.54	8.71 ± 0.59	7.76 ± 0.64	8.32 ± 0.49	7.88 ± 0.35	7.86 ± 0.60	8.31 ± 0.65	9.75 ± 0.73^a^	9.67 ± 0.88^a^

^*^AM ± SE, ^**^GM ± SE.

^a,b^Significantly different from individuals with CC and CT, respectively, *P* < 0.05.
